# Identifying factors influencing COVID-19 vaccine uptake in Finland – a qualitative study using social media data

**DOI:** 10.3389/fpubh.2023.1138800

**Published:** 2023-06-09

**Authors:** Anna-Leena Lohiniva, Annika Pensola, Suvi Hyökki, Jonas Sivelä, Vuokko Härmä, Tuukka Tammi

**Affiliations:** Finnish Institute for Health and Welfare, Helsinki, Finland

**Keywords:** COVID-19 vaccine, vaccine hesitancy, vaccine demand creation, behavioral insights, qualitative research, social media 2

## Abstract

**Introduction:**

Vaccine demand creation requires understanding what is driving the uptake of the vaccine. 24 Qualitative research methods are paramount to gaining a localized understanding of behavioral 25 drivers and barriers to vaccine uptake, but they are often underutilized.

**Methods:**

This is a qualitative study that 26 used public comments on the Facebook and Twitter posts of the Finnish Institute for Health and 27 Welfare (THL) as data sources to identify behavioral drivers for COVID-19 vaccine uptake in 28 Finland. The participatory data analysis utilized thematic analysis and the Theoretical Domains 29 Framework (TDF). NVIVO was used to assist in the coding process.

**Results:**

The greatest number of FB and 30 Twitter comments were linked with six TDF domains: knowledge, environmental context and 31 resources, beliefs in consequences, beliefs in capabilities, social and professional role, and social 32 influences. The domains included 15 themes that were interlinked. The knowledge domain 33 overlapped with all other domains.

**Discussion:**

By using public discourse on Facebook and Twitter, and rapid 34 qualitative data analysis methods within a behavioral insight framework, this study adds to the 35 emerging knowledge about behavioral drivers of COVID-19 vaccines that can be used by public 36 health experts to enhance the uptake of vaccines during future pandemics and epidemics.

## Introduction

The COVID-19 pandemic has triggered an intense focus on global research and development of COVID-19 vaccines which resulted in several vaccines being made available to the public within a year of the start of the pandemic (1). Vaccines are a key intervention to reduce pandemic-related mortality and morbidity (2, 3). Introducing a new vaccine is often challenging due to concerns among the public about its safety and efficacy, which can lead to vaccine hesitancy or refusal to take the vaccine. Vaccine hesitancy, which refers to a delay in acceptance or refusal of vaccination despite the availability of vaccination services, has been cited by the World Health Organization (WHO) as one of the top ten global health threats in 2019 (4, 5). Among other factors, such as convenience and complacency, vaccine hesitancy is also affected by the lack of confidence in vaccines and is sometimes fueled by conspiracy theories that are often perpetuated through social media channels (6, 7). Vaccine hesitancy is a complex and context-specific phenomenon that varies across time, place, and vaccine type (5, 8).

In Finland, the COVID-19 vaccination program began at the end of December 2020 for social and healthcare personnel with a higher risk of exposure, risk groups including those who have underlying health conditions and those aged 70 years or older, followed by all adults, then those 15 years or older and most recently for those 12 years and older. As of November 24, 2021, vaccine coverage was 76%; near the national vaccine coverage target of 80% (9). Repeated surveys on public perceptions of the COVID-19 vaccine in Finland indicate vaccine hesitancy has evolved during the pandemic but it has been relatively low overall. Concerns about side effects have been identified as one of the main reasons for vaccine hesitancy (10), and trust in the safety of the vaccine has been identified as the strongest predictor of COVID-19 vaccination intention (11).

Social media has become a source of data for understanding public attitudes and behaviors during emergencies (12–14). Large amounts of real-time data posted on social media platforms can be used to quickly identify public attitudes on issues of public health importance such as on COVID-19 vaccines to support health communication and health promotion messaging. A growing body of literature shows the use of social media platforms as data sources such as Twitter and Facebook for public health response and vaccine promotion (15, 16).

Qualitative research methods are paramount to exploring and understanding socially and culturally embedded vaccine behaviors (17). However, they are often underutilized with social media data as the content can be massive in volume and largely unrelated due to the dynamic nature of the online conversations, which makes it unpractical for qualitative research (18). Social media conversations can also be long with many users and no clear endpoint which makes identifying units of analysis difficult (19). Despite the challenges, social media data also provides many opportunities for qualitative researchers when compared to more traditional qualitative data collection methods such as in-depth interviews or focus group discussions. Social media data emerges from real-world social environments, without any prompting from researchers. Social media is a data source that can reach to individuals that may not be captured through traditional data collection methods. In addition, social media data can be collected rapidly and additional, if needed, can be easily obtained. (18).

To maximize vaccine uptake, it is critical to understand drivers of vaccine intention to design interventions and messages that best support vaccine uptake (20). Previous research has suggested that behavioral change interventions are more successful when they are grounded in theory and when they correspond with the concerns and perceptions of the target audience (21).

This paper describes a qualitative study that used social media as a data source and Theoretical Domains Framework (TDF) as a conceptual framework to identify and describe behavioral determinants in online COVID-19-related discussions. TDF consists of 14 domains that explain behavior including (1) knowledge, (2) skills, (3) social/professional role and identity, (4) beliefs about capabilities, (5) optimism, (6) beliefs in consequences, (7) reinforcement, (8) intentions, (9) goals, (10) memory, attention and decision processes, (11) environmental context and resources, (12) social influences, (13) emotion, and (14) behavioral regulation. TDF was selected because of its ability to help identify the barriers and facilitators to behavior change while taking into account social and environmental factors that drive behaviors (22). It has been widely used in various vaccine interventions (23). The findings of this research can be used to develop evidence-based interventions and messages for vaccine demand creation that correspond with the real needs and concerns of the public.

## Materials and methods

This is a qualitative study based on public comments on the Facebook and Twitter posts of the Finnish Institute of Health and Welfare (THL) from March 1, 2021 to May 31, 2021. The data was retrieved by using Emplifi, a social media management tool. All posts tagged with a “Corona” tag indicating COVID-19 as the main theme of the post were retrieved for further inspection. The specific subject matter of the COVID-19-related posts ranged from weekly updates on the pandemic situation to new related studies and to THL recommendations on topics such as mask use and remote working practices. From March–May 2021, THL made 367 Facebook and 546 Twitter posts, of which 214 and 316 were corona-tagged, respectively. As an official government entity, THL communicates in Finnish, Swedish and English. The majority of the posts are Finnish, and the few in other languages are translations of Finnish. Of the posts which were related to COVID-19 by subject matter, all posts that were not in Finnish and did not have at least one relevant comment in the Finnish language were discarded in the preliminary data screening. As the dataset consists of only Finnish comments, original posts in languages other than Finnish were therefore excluded. In addition, those posts which did not include comments were discarded as well.

After limiting the data to the preliminary requirements, the final dataset consisted of 144 Facebook and 123 Twitter posts. The posts collected from Facebook had 9,792 comments and 2,612 unique authors, while the Twitter posts had 932 replies and 420 unique authors. THL’s replies to comments and questions varied among posts and platforms but were nonetheless left in the data in order to preserve the context of the discussions in the comment section. Although the number of posts from both platforms is relatively equal, the tendency of a Facebook post to elicit interaction among followers is significantly higher. However, Socialbakers only allows for the collection of replies, not retweets.

The dataset was cleaned and anonymized manually by deletingtthe names of all private individuals along with any references to specific locations. All comments deemed irrelevant to the pandemic context and lacking a coherent message, such as recurring comments forcing the same joke or other bot-like behavior, were removed. The analysis was based on the TDF framework which consists of 14 domains of behavior that were adapted to the purpose of the study (24) by defining them *via* linkages to the COVID-19 vaccine. A total of 13 domains were included in the study. The domains and their definitions can be found in Table 1.

**Table 1:** TDF domains and their definitions adapted from (24).

TDF Domain	Definition
Knowledge and skills	Knowledge about the pandemic and/or COVID 19 vaccine
Beliefs about capabilities	Abilities to take the vaccine
Beliefs about consequences	Negative or positive outcomes as a result of having taken COVID19 vaccine
Environmental context and resources	Vaccine related actions of authorities, policies, procedures and resources
Goals	Decision to take vaccination
Emotions	Emotions linked with COVID-19 vaccine or COVID-19 vaccine uptake
Memory, attention, and decision processes	Influence of trust and mistrust towards COVID-19 vaccines, vaccination program, authorities COVID-19 response in decision-making process
Behavioral regulation	Having made a concrete action that indicates that COVID-19 vaccine either will or will not be taken
Social influences	Social influences related to COVID-19 vaccine, social norms, social pressures
Social and professional role	Group beliefs, perceptions, and behaviors linked with COVID-19 vaccines
Intention	Thinking of taking COVID-19 vaccine, having considered taking COVID19 vaccine
Optimism	Perception that taking COVID-19 vaccine will lead to some positive outcomes
Reinforcement	Perception of support, pressure, feedback that encourages uptake of COVID-19 vaccine

The analysis was a highly participatory multistage process between three members of the research team. It started by dividing the data among the team members who first read the narrative data independently to identify the type of domain or domains from a single comment or an entire discussion, followed by coding them into the appropriate domains using NVIVO software. The Finnish narrative was given codes in English by the research team members who all had proficiency in both languages. The team members met regularly to discuss and review the division of the data into different domains and the translation of the codes until they reached a consensus. The six TDF domains that received the most comments were included in the analysis. They included beliefs in consequences, environmental context and resources, knowledge, social and professional roles, social influences, and beliefs in capabilities. The total number of comments per domain can be seen in Table 2.

**Table 2:** Number of comments per TDF domain.

Theoretical Domain	No of comments
Environmental contest and resources	326
Beliefs about consequences	248
Knowledge	243
Social and professional roles and identity	142
Social influences	109
Total	1,068

A team of two continued the thematic analysis *via* an inductive coding process within each domain separately to allow new ideas and concepts to emerge freely from the data (25). The process included developing codes, subcategories, and themes for each domain (26). Once the domains were analyzed the team members shared their themes in a joint platform to discuss the themes and their linkages. Any discrepancies in coding and the themes were discussed until consensus was reached. In the final stage, the team members jointly reviewed the themes across the domains and their connections to come up with a final interpretation to explain the domains. Only saturated themes were included in the final interpretation of the data.

The study protocol was approved by the Institutional Review Board of the Finnish Institute for Health and Welfare in February 2021.

## Results

**Figure 1:**
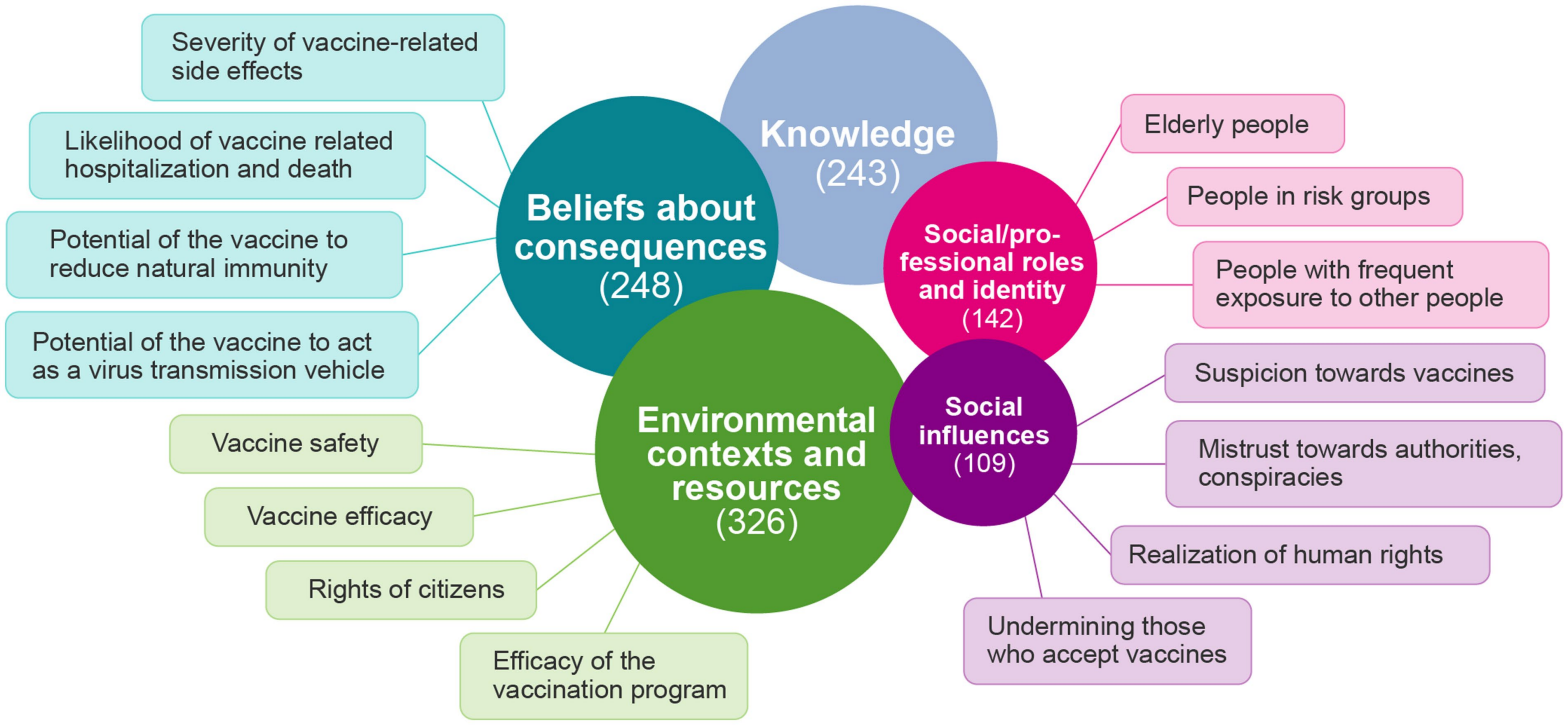
Behavioral domains and themes linked with vaccine uptake.

The section describes five TDF domains that were identified as having the greatest number of comments on both FB and Twitter. The section starts with the description of knowledge and beliefs in consequences followed by environmental context and resources, social and professional roles, social influences, and beliefs about capabilities. Figure 1 shows the domains and linked themes.

### TDF domain: knowledge

Comments that reflected questions overlapped with all themes of all four domains described earlier. In addition, questions linked with TDF domain beliefs about capabilities emerged from the analysis including the physical capability to take the vaccine. That included questions about vaccine eligibility based on personal attributes such as illnesses, medical conditions, and prior medical procedures.

*“What type of vaccine will I receive based on my health conditions and age?”*

### TDF domain: beliefs in consequences

Five themes were identified describing the beliefs in consequences related to COVID-19 vaccine uptake: severity of vaccine-related side effects, the likelihood of vaccine-related hospitalization and death, the potential of the vaccine to reduce natural immunity, potential of the vaccine to act as a virus transmission vehicle, and the utility of the vaccine.

#### Theme 1: severity of vaccine-related side effects

Comments about the vaccine-related severity of the side effects were divided between those that referred to severe symptoms and illness episodes and those that emphasized the mild nature of the side effects. The comments were either experiences of the commentators, those of their social network, or general statements and hypothetical questions most commonly without reference to any study or authority. Questions without a personal viewpoint often related to how common certain side effects were among the population.

Comments about mild symptoms included slight fever, fatigue, diarrhea, and headache, among others. They were often posted to defend vaccine uptake or debate against comments that promoted fear towards vaccine-related side effects. Some comments referring to side effects as normal characteristics of any vaccination were posted in the discussions to encourage others to take the vaccine.

*“Vaccinations and medications in general always have side effects.”*

Comments about strong side effects included severe fever, shortness of breath, and pain. The comments were typically posted to discourage vaccine uptake. In a few comments, respondents noted that they were not planning to get the vaccine or the second shot because of the side effects.

*“I have heard so much about side effects that I rather not take the vaccine.”*

#### Theme 2: likelihood of vaccine-related hospitalization and death

Most of the comments about vaccine-related hospitalization and death questioned vaccine-related mortality and morbidity. Some comments raised concerns and suspicions that authorities may be hiding vaccine-related hospitalization and deaths. Some comments were firm statements that COVID-19 vaccines cause death. Most of these comments did not include sources to support the statements.

*“How many people who have taken the corona vaccine have ended up in the hospital?”*

#### Theme 3: potential of the vaccine to reduce natural immunity

Comments also included concerns about losing natural immunity when taking the COVID-19 vaccine, which was frequently linked with mNRA vaccines in the comments. For example, comments reflected beliefs that the vaccines contained gene manipulating features that may weaken individuals physically or mentally or concern about the poor quality of the vaccines because of the rapid vaccine development process.

“*Vaccines reduce immunity. Imagine what other things can happen when you take a vaccine that manipulates your genes. I cannot even begin to think about it.”*

#### Theme 4: potential of the vaccine to act as a virus transmission vehicle

Some comments reflected concern that vaccines were transmitting the virus instead of curbing the pandemic. This was explained in multiple ways. For example, because the vaccine contained the virus, it was being spread through the vaccination program itself. Some comments highlighted that vaccinated individuals had the potential to infect non-vaccinated individuals with the virus when in close contact such as on public transportation or in the workplace. Other comments referred to COVID-19 vaccines as a mode of transmission for new virus strains.

*“Vaccines contain the virus. The more vaccines the more virus we have around.”*

#### Theme 5: the utility of the vaccine

Some comments referred to vaccines as useless because even after vaccination one had to continue adopting prevention measures such as wearing a mask. Other comments highlighted disappointment that the vaccination did not reduce the risk of transmission of the virus. Additional questions were raised about how useful the COVID-19 vaccines were against different variants and in combination with illnesses and other factors influencing one’s personal health. Other comments noted that vaccines are useless because new virus strains are emerging, and vaccination programs were seen as slow and insufficient to cover the entire population.

*“What is the benefit of the vaccine? We keep taking our precautions when meeting people and using masks. Nothing changes.”*

Comments about the benefits of the vaccine typically highlighted the ability of the vaccine to prevent severe illness or to help reach herd immunity which is necessary to halt the pandemic.

*“At least you do not get really ill from the virus if you have taken the vaccine.”*

### TDF domain: environmental context and resources

The domain included comments that were directed to authorities involved with the national vaccination programs and vaccination resources. Four themes emerged from the analysis: the safety of the vaccines, the efficacy of the vaccines, the rights of the citizens, and the efficiency of the vaccination program.

#### Theme 1: safety of the vaccines

Many vaccine safety-related comments related to the investigations into vaccine side effects globally and in Finland. Many comments also questioned the system and reliability of reporting side effects.

*“Reporting side effects is voluntary. How do we know how many people have actually had side effects? Nobody knows the real situation*.”

Vaccine safety-related comments also questioned authorities’ decisions regarding the vaccination order and their ability to protect the most vulnerable with the vaccination order. In addition, vaccine safety comments included concerns about the authorities’ decisions to mix of different vaccine types. Some comments called for more investigations into how the mixing of different types of vaccines impacted vaccine safety and others wanted to understand the reasoning behind the decision. Many comments also related to the ability and reasoning of the authorities to stop the distribution of vaccines if they were identified as unsafe.

*“Other countries have stopped the entire vaccine program until they know more about the recent episodes with blood clots. Why is Finland not taking the same action?”*

#### Theme 2: efficacy of the vaccines

Efficacy-related comments typically questioned the decisions and actions of the authorities regarding the inclusion of vaccines by certain manufacturers, mixing of vaccines of different manufacturers’, or defining the time between the vaccine shots. In addition, many comments discussed the strengths of the available vaccines against new virus strains.

*“I do not know how they know what the impact is of mixing these two vaccines. I am not convinced at all.”*

#### Theme 3: rights of the citizens

Comments also frequently reflected the rights of the citizens to choose to comply with the vaccination order of the government that prioritized at-risk groups, the type of vaccine, the right to be informed about the type of vaccine and the right to decide not to be vaccinated.

*“We should be informed about different types of vaccines and side effects and everything else. Nobody should be forced to take the vaccine.”*

#### Theme 4: efficiency of the vaccination program

A number of comments related to vaccination logistics, such as the time to get vaccinated, the vaccination schedule, and the time vaccination took place. Some comments related to attitudes and the service of healthcare personnel at the vaccination location. Many of these comments were based on own experiences and most of them were positive.

However, some comments also noted problems such as difficulties scheduling the vaccination appointment or lack of information about the type of vaccine they received. Some comments included concerns about the capability of the vaccinators to assess the physical abilities of the individuals to take the vaccine.

*“It is not easy to find out how to book and where to book your vaccination.”*

Some comments reflected concerns about the political nature of decision-making regarding the vaccination program. Particularly, the geographic distribution of the vaccines generated equity-related discussions.

*“Why do some areas (districts) get more vaccines than other areas (districts)*? *It does not sound right.”*

The comments included questions about vaccine coverage to understand how many people had been vaccinated in Finland which often was used to evaluate the success of the vaccination program. The national distribution of the vaccines was questioned when commentators did not understand the regional distribution system or when they wanted to point out that the system was unfair. Comments related to the vaccination order often questioned the fairness of the current distribution plan across different population sections and geographic locations. Questions were also raised about the vaccination interval between the two doses to understand the variation.

### TDF domain: social and professional roles and identity

The domain described groups of people who were seen as at risk for COVID-19, which included people in risk groups, the older adult, and those frequently exposed to other people.

#### People in risk groups

The comments frequently referred to risk groups which included various groups of people with underlying health conditions such as those with diabetes, obesity, heart problems, or hypertension. Risk groups were referred to as vulnerable populations and as a priority population for the COVID-19 vaccine.

*“I am in a risk group and still waiting [for the vaccine].”*

#### Older adult people

The comments also noted age as a risk factor for COVID-19. The most substantive individual age range mentioned in the comments was that of 59-65-year-olds. Age was often linked with a specific health condition. On the contrary, young age was seen as protection against the virus. Many comments highlighted that young people did not need to be prioritized in the vaccination program.

*“When am I eligible for vaccination? I had corona at the beginning of the year, I am 69 years old and I have asthma.”*

#### Those frequently exposed to other people

The comments referred to people in certain professions such as caretakers or personal assistants for the older adult, those in the service industry, or healthcare personnel as at risk of contracting COVID-19. The comments also referred to people whose lifestyle exposed them to COVID-19 such as those who travel or like to gather together and socialize with other people.

*“How is it possible that all the dental personnel is not vaccinated?”*

### Domain: social influences

The domain describes the views and ideas of other groups of people about COVID-19 vaccines. Four themes were identified: suspicion towards the vaccines, undermining those who have taken the vaccine, mistrust towards the authorities and conspiracies, and realization of human rights.

#### Theme 1: suspicion towards the vaccines

The comments reflecting suspicion towards COVID-19 vaccines included arguments that the vaccines are experimental and humans are test rabbits. Some comments referred to the vaccines as poison due to the fast-track manufacturing process. A substantial portion of these comments were short statements rather than narratives with a rationale for the belief.

*“It’s an experimental vaccine, and the companies are free from responsibility. It’s a worldwide human experiment.”*

#### Theme 2: undermining of people who accept to get vaccinated

Many comments reflected criticism towards those who took the vaccine. The “do your own research” rhetoric was present in comments condemning people as brainwashed and falling for a scam by getting vaccinated.

*“By getting vaccinated you indicate that you have no survival instinct.”*

#### Theme 3: mistrust towards the authorities and conspiracies

The comments included blame towards public health and government officials for deliberately hiding information on vaccine manufacturing procedures and statistics on deaths and side effects. From some comments, it was evident that vaccines were seen as a tool for population control and for financial benefits at the expense of the population.

*“Vaccines are made to reduce the population of the world.”*

#### Theme 4: realization of human rights

Several discussions raised concerns about equality among the vaccinated and unvaccinated in terms of their freedom of movement, and their ability to join various functions and participate in public events and activities.

*“Soon we will not have the right to buy any goods or services unless we have been vaccinated with this experimental vaccine.”*

## Discussion

Our study provided important insights into COVID-19 vaccine-related online discussions and demonstrated that using rapid qualitative data analysis methods with social media data allows research teams to gain insights into vaccine-related barriers and facilitators. The established system can also be reinitiated at different times to monitor potential changes in the barriers and facilitators.

The study showed that the discussions of different domains were in many ways linked. The domain knowledge was of particular importance as it overlapped with all other domains. This was evident from a large number of questions posted in the comments on both FB and Twitter across all themes identified in this study. The large number of questions can be partially explained by the fact that THL is one of the national authorities providing guidance during the pandemic and accordingly people seek answers to their questions on THL’s social media platforms. On the other hand, the number of questions shows that the public is in need of updated and understandable information about vaccines, which has been identified as a gap also in other countries such as Canada and in Spain (27, 28). As the public in Finland received information during the pandemic from a number of different entities that communicate to the public independently including the Ministry of Health at the central and local levels, municipalities, THL, and others, the number of questions may be due to confusion. It is important that the concerns and the questions of the public are captured and answered in real-time to avoid an infodemic meaning an overabundance of information—some accurate and some not—that occurs during an epidemic, has been a challenge globally (29). The methodology that we used in the study could be used to develop a social listening system to monitor changes in barriers and facilitators with a special focus on trending questions and concerns. Social listening is being increasingly used to monitor public opinion, risk perceptions, and concerns during the pandemic globally (15, 30).

The findings of our study also showed that vaccine safety and efficacy were a major concern for members of the public who wanted reassurance from the authorities that the vaccines being provided by the government health authorities were safe and efficient, which aligns with other recent COVID-19 vaccine-related studies conducted in other countries in the world that show COVID-19 vaccination intentions being strengthened through a simple messaging intervention that utilizes perceived vaccine response efficacy (31–34). In addition, our study indicated that the public was also concerned about citizens’ rights in the midst of the fast-phased vaccination campaigns and the overall capabilities of the authorities to manage such a massive undertaking, which has also been discussed widely in countries around the world (35, 36).

We learned through the study that beliefs in the consequences of taking the COVID-19 vaccine included comments about side effects and vaccine-related hospitalization and death, which were often bound to the experiences of the commenters. Those who mentioned severe symptoms aimed to convince others not to take the vaccine whereas those who referred to mild side effects aimed to encourage vaccine uptake. As sharing own experiences online has been identified as a powerful technique to get messages across and even change behavior (37), risk communication may benefit from the personal testimonials that the public shares. For example, mild experiences could be used to promote vaccine safety (38).

Our study also showed that groups of people who were seen at risk for COVID-19 were those who were perceived as a risk group due to some underlying health conditions, the older adult, and those who were exposed to frequent contact with other people, which highlights the challenges that risk communicators can face when aiming to convince other target audiences such as young people to take the vaccine. This highlights the need for targeted communication campaigns that use tailored interventions with different target audiences to motivate vaccine uptake. It further highlights the importance of context-specific behavior change interventions that translate global strategies to local approaches (20). Recent COVID-19 message frame experiments in other countries further demonstrate the need for context specificity and highlight the importance of identifying the appropriate message frames for different settings and different audiences for each context (39, 40).

We had limitations in our study. It was not possible to obtain much information about the background characteristics of those who comment on FB and Twitter, which makes the generalization of the results or the development of targeted communication messaging for various sub-populations, such as age groups challenging. The use of diagnostic queries in the analysis should be considered and tested in the future to better capture the activity of specific peoples, places, events, and times.

## Conclusion

By using a behavioral insight framework, this study adds to the emerging knowledge about public perceptions of COVID-19 vaccines by analyzing public discourse on FB and Twitter posts. Health authorities can use this knowledge to develop vaccine-demand interventions that are responsive to the concerns of the public. The methodology can be also scaled up and used over time to monitor changes in vaccine-related barriers and facilitators in real-time.

## Data availability statement

The data analyzed in this study is subject to the following licenses/restrictions: Raw (anonymized) data will be made available on reasonable request with any data that may risk the loss of confidentiality redacted. Requests to access these datasets should be directed to Further inquiries can be directed to the corresponding author.

## Ethics statement

The studies involving human participants were reviewed and approved by the Ethical Board of the Finnish Institute for Health and Welfare. Written informed consent for participation was not required for this study in accordance with the national legislation and the institutional requirements.

## Author contributions

A-LL: conceptualization, methodology, analysis, and writing original draft. AP: conceptualization, methodology, analysis, writing, review, and editing. SH: conceptualization, methodology, analysis, writing, review, editing, and visualization. JS: conceptualization, funding acquisition, administration, writing, review, and editing. VH: administration, validation, writing, review, and editing. TT: conceptualization, funding acquisition, supervision, writing, review, and editing. All authors contributed to the article and approved the submitted version.

## Funding

This research was funded by the Academy of Finland.

## Conflict of interest

The authors declare that the research was conducted in the absence of any commercial or financial relationships that could be construed as a potential conflict of interest.

## Publisher’s note

All claims expressed in this article are solely those of the authors and do not necessarily represent those of their affiliated organizations, or those of the publisher, the editors and the reviewers. Any product that may be evaluated in this article, or claim that may be made by its manufacturer, is not guaranteed or endorsed by the publisher.
